# Multi-channel lung sounds intelligent diagnosis of chronic obstructive pulmonary disease

**DOI:** 10.1186/s12890-021-01682-5

**Published:** 2021-10-15

**Authors:** Hui Yu, Jing Zhao, Dongyi Liu, Zhen Chen, Jinglai Sun, Xiaoyun Zhao

**Affiliations:** 1 Academy of Medical Engineering and Translational Medicine, Tianjin University, Tianjin, 300072 China; 2 Department of Biomedical Engineering, Tianjin Key Laboratory of Biomedical Detecting Techniques and Instruments, Tianjin University, Tianjin, 300072 China; 3 Chest Hospital of Tianjin University, Tianjin, 300051 China

**Keywords:** Hilbert–Huang tansform, ReliefF algorithm, RespiratoryDatabase @ TR, Chronic obstructive pulmonary disease

## Abstract

**Background:**

Chronic obstructive pulmonary disease (COPD) is a chronic respiratory disease that seriously threatens people’s health, with high morbidity and mortality worldwide. At present, the clinical diagnosis methods of COPD are time-consuming, invasive, and radioactive. Therefore, it is urgent to develop a non-invasive and rapid COPD severity diagnosis technique suitable for daily screening in clinical practice.

**Results:**

This study established an effective model for the preliminary diagnosis of COPD severity using lung sounds with few channels. Firstly, the time-frequency-energy features of 12 channels lung sounds were extracted by Hilbert–Huang transform. And then, channels and features were screened by the reliefF algorithm. Finally, the feature sets were input into a support vector machine to diagnose COPD severity, and the performance with Bayes, decision tree, and deep belief network was compared. Experimental results show that high classification performance using only 4-channel lung sounds of L1, L2, L3, and L4 channels can be achieved by the proposed model. The accuracy, sensitivity, and specificity of mild COPD and moderate + severe COPD were 89.13%, 87.72%, and 91.01%, respectively. The classification performance rates of moderate COPD and severe COPD were 94.26%, 97.32%, and 89.93% for accuracy, sensitivity, and specificity, respectively.

**Conclusion:**

This model provides a standardized evaluation with high classification performance rates, which can assist doctors to complete the preliminary diagnosis of COPD severity immediately, and has important clinical significance.

## Background

COPD is a common preventable and treatable disease characterized by continuous airflow restriction. Airflow restriction develops progressively, related to the increased chronic inflammatory response to toxic particles or gases in the airway and lungs. COPD has high morbidity and mortality worldwide and has become the fourth leading cause of death in China and the third leading cause of death globally [1]. According to the global initiative for chronic obstructive lung disease (GOLD), in 2020, the prevalence of COPD was 11.7%, and there were approximately 3 million deaths each year. As populations age in high-income countries and the increase of smokers in developing countries, it is roughly calculated that by 2060, over 5.4 million people will die each year from COPD and related diseases [2].

The severity of COPD is graded according to FEV1/FVC, FEV1%, and symptoms [3]. FEV1 refers to the forced expiratory volume in one second, FVC refers to the forced vital capacity. FEV1/FVC is a sensitive index to evaluate airflow limitation, and FEV1% is a good indicator to evaluate COPD severity. The severity of COPD is classified into five grades by GOLD. Patients with COPD0 have FEV1% higher than 85%, COPD1 have FEV1% higher than 80%, COPD2 have FEV1% between 50% and 80%, COPD3 have FEV1% between 30% and 50%, COPD4 have FEV1% less than 30%, or less than 50% but suffer from chronic respiratory failure. In addition, FEV1/FVC is less than 70% in all patients except patients with COPD0.

However, studies have shown that the association between patients’ health status, symptoms, and FEV1 is weak [4, 5]. In addition, the number of classifications obtained by this way is excessive, which is rarely used in clinical practice. Through communication with doctors, it is known that the severity of COPD is often classified into three grades of mild, moderate, and severe in clinical practice, and then the specific diagnosis and treatment plan was determined according to specific symptoms. Generally speaking, mild patients will get better after taking tracheal relaxants, moderate patients will decide outpatient or hospitalization according to specific symptoms, and severe patients should be hospitalized immediately or even need ICU rescue. At present, the clinical diagnosis of COPD includes pulmonary function examination, chest X-ray examination, chest CT examination, blood gas examination, and other diagnostic methods [6–8]. These methods are time-consuming, invasive, and radioactive, unsuitable for daily screening. Hence, it is urgent to develop a non-invasive and rapid COPD severity diagnosis technique suitable for daily screening.

Lung sound, as a physiological sound signal produced in the process of gas exchange between the human body and the outside world, contains a lot of physiological and pathological information, representing the health status of the human respiratory system. Pulmonary auscultation plays an essential role in the diagnosis of respiratory diseases and their severity. Previous studies have shown that pulmonary auscultation can be used as an index for preliminary diagnosis of COPD and its severity, worthy of clinical promotion and application [9–15]. The traditional artificial auscultation method requires experienced respiratory doctors and is limited by environmental factors. Therefore, the diagnosis technology of COPD based on lung sounds is of great significance to clinical practice and research, provides basic theory and experience for further development of diagnostic equipment for COPD.

Previously, most studies have used multi-dimensional features in the diagnosis of COPD. For example, QianWang et al. used the transfer learning algorithm based on balanced probability distribution and instances to diagnose COPD, and the accuracy rate reached 95.2% [16]; Jun Ying et al. utilized DBN to predict the exacerbation frequency of COPD, and the accuracy reached 91.99% [17]. Some studies diagnose COPD based on lung sounds, extract features through the short-time Fourier transform, wavelet transforms, or HHT, and then use an artificial neural network or deep learning algorithm for recognition and classification, achieving high accuracy. For instance, Morillo et al. inputted the features of lung sounds extracted by short-time Fourier transform into an artificial neural network classifier to identify COPD, and an accuracy of 81.8% has been achieved [18]. Altan et al. used the 3D mapping technique to extract features and the DBN classifier model to separate the severity of two COPD, which is utilized for preliminary diagnosis of COPD, with an accuracy of 95.84% [19]. Based on the statistical characteristics of HHT of lung sounds, Altan et al. used DBN to separate COPD patients from healthy subjects with an accuracy of 93.67% [20]. Altan et al. used the 3D second-order difference plot to extract characteristic abnormalities on lung sounds and then used the deep extreme learning machines classifier to classify the severity of COPD, with an accuracy of 94.31% [21]. Ahmet extracted the features of lung sounds through empirical wavelet transform and then input them into many models to distinguish COPD patients from healthy subjects [22]. Altan et al. used HHT to extract the features of lung sounds and fed the feature set into the proposed Deep ELM with HessELM-AE to distinguish COPD patients from healthy subjects, achieving an accuracy rate of 92.22% [23].

However, there are still some problems in the current research. These methods using multi-dimensional features require much information, including lung function, health status, BODE index, and risk assessment based on the GOLD. The process of collecting information is time-consuming and requires experienced respiratory doctors, making it difficult to make a preliminary diagnosis of COPD quickly. Due to doctors’ experience, human ear resolution, and environmental factors, it is challenging to diagnose the severity of COPD quickly and accurately. The current diagnosis based on lung sounds primarily focuses on separating COPD patients from healthy subjects and classifying COPD0 and COPD4, ignoring the clinical needs. Although few studies have completed five classifications of COPD severity, this classification method is rarely used in clinical practice. In addition, these researches adopt full-channel lung sounds, which increases the difficulty of collection and calculation. Moreover, it will lead to over-fitting the model [24] and be challenging to achieve a rapid and real-time diagnosis, reducing the practicability and portability of future diagnosis equipment and limiting the development of online diagnosis tools.

In this paper, firstly, the time-frequency-energy features of 12-channel lung sounds were extracted by HHT. Then, channels and features were selected by the reliefF algorithm to simplify the collection process and the number of features. Finally, the feature sets were input into SVM, Bayes, decision tree, and DBN to separate the auscultation records of mild, moderate, and severe COPD patients. This model can complete the preliminary diagnosis of COPD severity in a short time through the 4-channel lung sounds, considerably shortening the diagnosis time and saving the cost by canceling some additional diagnostic tools, which have important clinical significance.

The main contributions of this paper are as follows:

1. This study focuses on diagnosing COPD severity, categorizing it into clinically common grades: mild COPD, moderate COPD, and severe COPD. The model proposed in this study is able to reduce the diagnosis time, save medical resources, and assist doctors in completing the preliminary diagnosis of COPD severity. This model has been recognized by respiratory doctors in Chest Hospital of Tianjin University and Tianjin Medical University General Hospital in China.
2. The channel selection model based on the reliefF algorithm is used to determine the optimal channels for diagnosing the severity of COPD, which proves that use only 4-channel lung sounds of L1, L2, L3, and L4 channels that can undertake the diagnosis of COPD severity without 12-channels. It reduces acquisition difficulty and calculating pressure, contributing to developing portable diagnostic equipment for COPD.

The rest of this paper is structured as follows: The materials and methods are briefly introduced in “Materials and methods” section. “Experimental results” section provides the experimental process and results in detail. “Discussion” section presents the discussion. The conclusions of this paper and the following work are described in “Conclusion” section.

**Table 1:** The distribution of COPD patients by gender

	COPD0	COPD1	COPD2	COPD3	COPD4
Male	4	4	7	6	13
Female	2	1	0	1	4

**Fig. 1:**
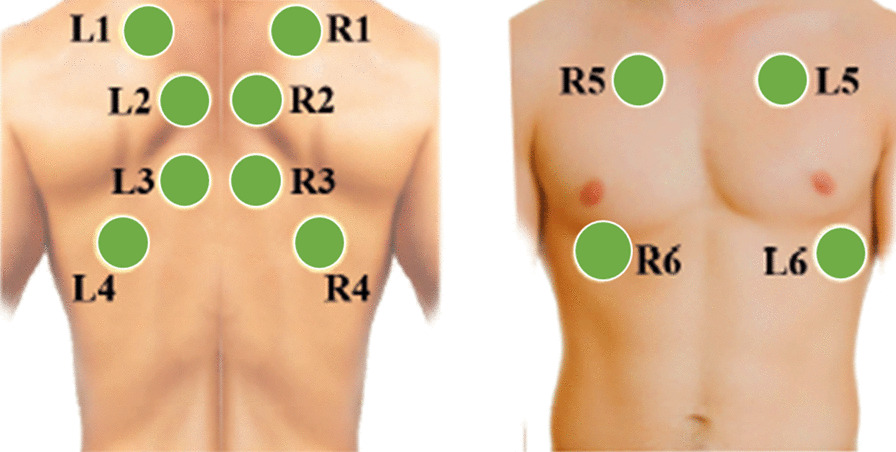
Lung auscultation areas

**Fig. 2:**
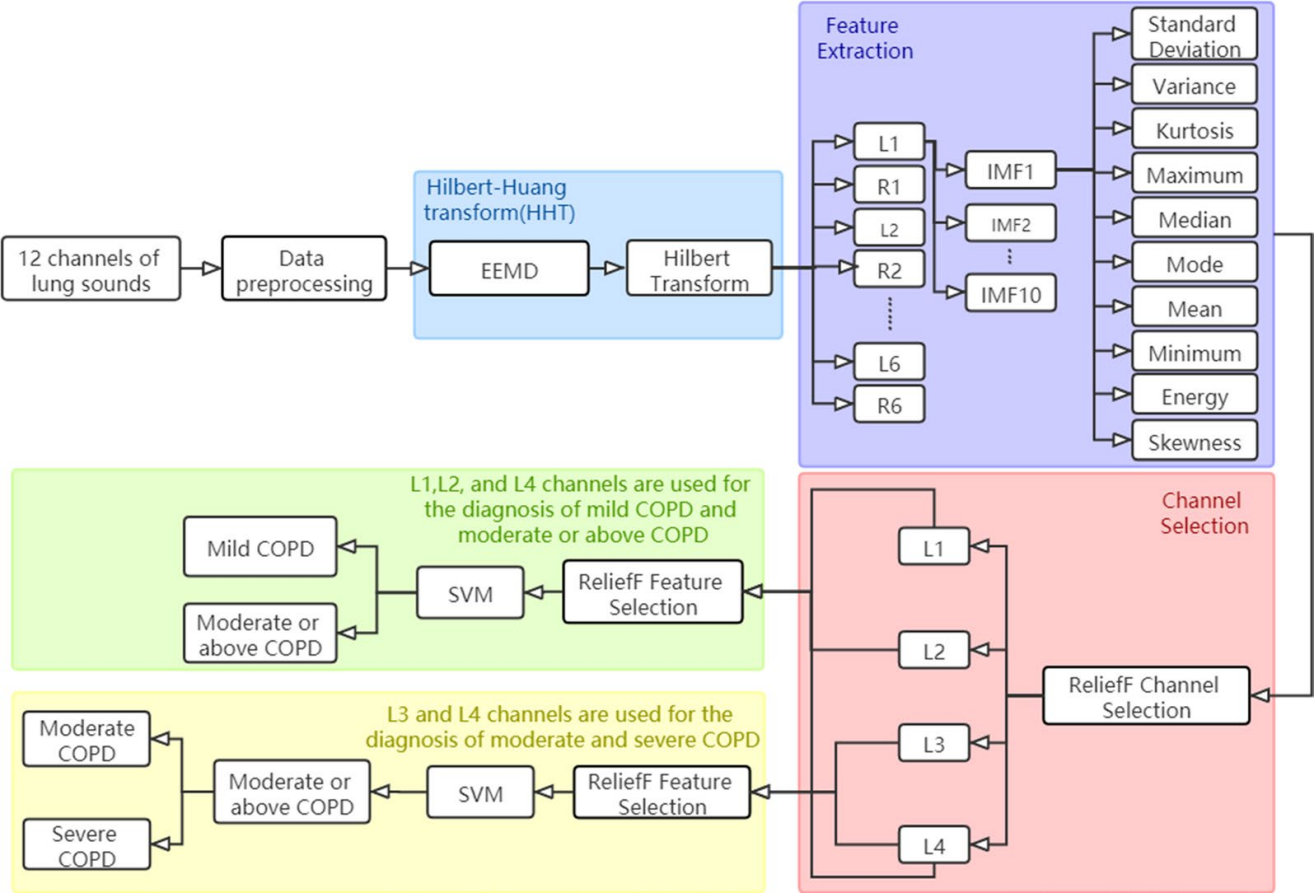
The technical architecture of our model

**Fig. 3:**
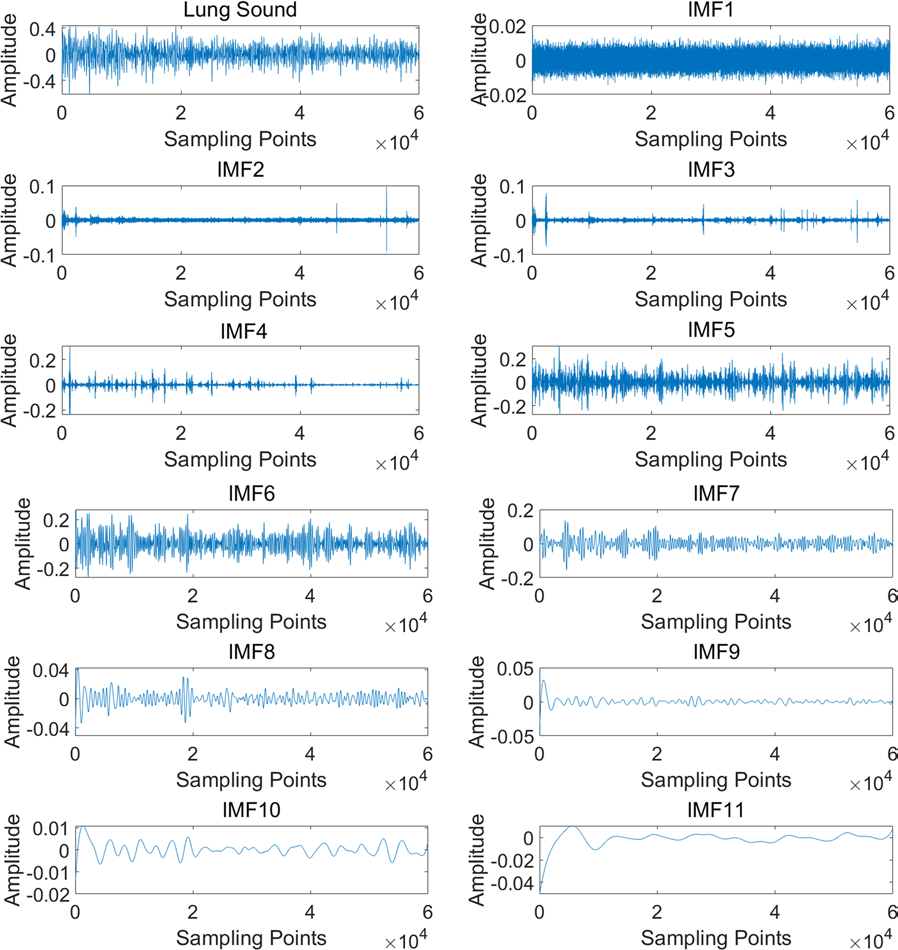
The IMFs obtained by using EEMD to a lung sound with COPD

## Materials and methods

### Database

As a public multimedia respiratory database, RespiratoryDatabase@TR contains 12-channel lung sounds, 4-channel heart sounds, spirometry metrics, and chest X-rays for each subject [25]. The respiratory data were obtained by two pulmonologist clinicians using a Littmann3200 digital stethoscope to record the left (L) and right (R) channels in each lung region simultaneously. The sampling frequency is 4000Hz, and lung auscultation areas are shown in Fig. 1.

The database contains lung sounds of 42 COPD patients with varying degrees of severity, ranging in age from 38 to 68, including 34 males and 8 females. The distribution of COPD patients by gender is shown in Table 1. Subjects had never used cigarettes or tobacco products, and none of their close relatives had asthma or COPD [25]. At the start of each recording, subjects were required to cough for the signal synchronization of each lung region’s left and right channels. The duration of each recording was at least 17s. The labeling of respiratory data was determined by two pulmonologist clinicians based on the COPD severity rating given by the GOLD. RespiratoryDatabase@TR has an ethical committee approval confirmed by Mustafa Kemal University, Turkey.

### Technical architecture

The proposed model in this paper contains four main parts: data preprocessing, feature extraction, channel selection, and COPD severity diagnosis. The technical architecture of our model is shown in Fig. 2. Firstly, the 12-channel lung sounds are preprocessed by segmentation and denoising, and the data is enhanced by adding noise. Then, the multi-dimensional features are extracted by HHT based on ensemble empirical mode decomposition (EEMD). Next, the channels and features are filtered based on the reliefF algorithm. Finally, SVM is used to diagnose the severity of COPD, which is classified into mild COPD, moderate COPD, and severe COPD.

### Data preprocessing

31 COPD patients with different severity were randomly selected from RespiratoryDatabase@TR (6 patients with COPD0, 5 patients with COPD1, 5 patients with COPD2, 5 patients with COPD3, 10 patients with COPD4). Combined with the professional advice of clinicians, it is necessary to auscultation two or three respiratory cycles in each auscultation site for pathological significance to realize the objective diagnosis. Hence, lung sounds were divided into 15 seconds with 60000 sample points in each segment for subsequent analysis, and the start point of segmentation was set as the first inhalation after the coughing peak [20]. Patients with COPD usually produce continuous and persistent abnormal lung sounds with a frequency above 400Hz [26]. The low-pass filter was abandoned to avoid losing important information, and only a 7.5Hz first-order Butterworth high-pass filter was used to eliminate DC offset.

According to the classification of COPD severity in clinical practice, COPD0 and COPD1 are called mild COPD, COPD2 and COPD3 are combined as moderate COPD, and COPD4 is severe COPD. The unbalanced sample size will lead to the poor training effect of the model and can not truly reflect the accuracy and recall rate of positive and negative samples. Therefore, to ensure a balanced sample size and facilitate subsequent classification, the respiratory data of COPD0 patients were increased to 10 groups by adding noise. The noise was Gaussian white noise with a mean of 0 and a standard deviation of 1; the noise factor was set at 0.06.

### Hilbert–Huang transform

HHT is a new nonstationary and nonlinear signal analysis method proposed by Huang E after an in-depth study and summary of previous signal analysis methods [27], which has been widely used in the model research of disease diagnosis, such as chronic respiratory diseases [20, 22, 28] and heart diseases [29]. The method mainly includes empirical mode decomposition (EMD), Hilbert transform (HT), and its spectral analysis, among which EMD is the core part of the algorithm [30].

EMD is a method of adaptively decomposing the signal into a series of intrinsic mode functions (IMFs) based on its characteristics. The implementation steps are as follows:

1. Calculate all local extremum points (including maximum and minimum points) of this signal, and fit them respectively to get the upper (${\mathrm {f}}_{\max }({\mathrm {t}})$) and lower (${\mathrm {f}}_{\min }({\mathrm {t}})$) envelopes with the cubic spline interpolation algorithm.
2. The average values of the upper and lower envelopes are calculated and used as the mean envelope n(t) of the original signal:
  1 $$\begin{aligned} {\mathrm {n}}({\mathrm {t}})=\frac{{\mathrm {f}}_{\max }({\mathrm {t}})+{\mathrm {f}}_{\min }({\mathrm {t}})}{2} \end{aligned}$$
3. Subtract the mean envelope n(t) from the unprocessed signal s(t):
  2 $$\begin{aligned} m_{1}({\mathrm {t}})={\mathrm {s}}({\mathrm {t}})-{\mathrm {n}}({\mathrm {t}}) \end{aligned}$$
4. Determine whether $m_{1}({\mathrm {t}})$ is an IMF. If so, ${\mathrm {c}}_{1}({\mathrm {t}})=m_{1}({\mathrm {t}})$. If not, re-do the analysis of $1) \sim 3)$ based on this signal until $m_{1}({\mathrm {t}})$ is an IMF.
5. After obtaining the first IMF using the above method, subtract ${\mathrm {c}}_{1}({\mathrm {t}})$ from the original signal s(t) to get the residual component ${\mathrm {r}}_{1}({\mathrm {t}}):$
  3 $$\begin{aligned} {\mathrm {r}}_{1}({\mathrm {t}})={\mathrm {s}}({\mathrm {t}})-{\mathrm {c}}_{1}({\mathrm {t}}) \end{aligned}$$
6. Let ${\mathrm {r}}_{1}({\mathrm {t}})$ as the new original signal, IMF2 can be obtained through the analysis of $1) \sim 5)$, and so on until the predetermined stop criterion is met. The EMD decomposition is completed.

Finally, the original signal s(t) is broken down the sum of a residual component and a series of IMFs:

4 $$\begin{aligned} {\mathrm {s}}({\mathrm {t}})=\sum _{{\mathrm {i}}=1}^{{\mathrm {n}}} {\mathrm {c}}_{{\mathrm {i}}}({\mathrm {t}})+{\mathrm {r}}_{{\mathrm {n}}}({\mathrm {t}}) \end{aligned}$$

EMD has good properties, including adaptability, completeness, and approximate orthogonality, but it also has some shortcomings, the most important of which is the mode-mixing problem. In order to avoid this phenomenon, EEMD was used to decompose lung sounds. EEMD is essentially a multiple EMD with Gaussian white noise. Gaussian white noise has the characteristic of uniform spectrum distribution, so adding it to the signal will automatically separate the signals of different time scales into the corresponding reference scale. The white noise is canceled by averaging the corresponding IMFs obtained by multiple EMDs [31].

The HT of the signal is the output response $x_{h}(t)$, which is of a continuous-time signal x(t) through a linear system with the impulse response ${\mathrm {h}}({\mathrm {t}})=\frac{1}{\pi t}$. The physical meaning of HT is to postpone all frequency components’ phrases by 90 degrees.

The analytic function of x (t) ’s HT:

5 $$\begin{aligned} {\mathrm {H}}(\omega , {\mathrm {t}})={\text {Re}} \sum _{{\mathrm {i}}=1}^{{\mathrm {n}}} {\mathrm {c}}_{{\mathrm {i}}}({\mathrm {t}}) {\mathrm {e}}^{{\mathrm {j}} \theta ({\mathrm {t}})}={\text {Re}} \sum _{{\mathrm {i}}=1}^{{\mathrm {n}}} {\mathrm {c}}_{{\mathrm {i}}}({\mathrm {t}}) {\mathrm {e}}^{{\mathrm {j}} \int \omega _{{\mathrm {i}}}({\mathrm {t}}) {\mathrm {dt}}} \end{aligned}$$

Re indicates the real part, $c_{i}(t)$ indicates the instantaneous amplitude, and $\omega _{i}(t)$ indicates the instantaneous frequency. By integrating the time, the Hilbert marginal spectrum was obtained:

6 $$\begin{aligned} {\mathrm {h}}(\omega )=\int _{0}^{{\mathrm {T}}} {\mathrm {H}}(\omega , {\mathrm {t}}) {\mathrm {dt}} \end{aligned}$$

It accumulates the amplitudes of each frequency, showing the global contribution of each frequency.

By squaring the amplitude of the Hilbert marginal spectrum and integrating the frequency, the instantaneous energy density IE (t) was obtained:

7 $$\begin{aligned} {\mathrm {IE}}({\mathrm {t}})=\int _{\omega _{1}}^{\omega _{2}} {\mathrm {H}}(\omega , {\mathrm {t}})^{2} {\mathrm {d}} \omega \end{aligned}$$

### ReliefF feature selection

Since scale differences among features will affect the selection of valuable features, and the subsequent classification algorithm also requires data normalization, the feature sets of samples were normalized. This paper used the Mapminmax function in Matlab to normalize the feature sets to [0,1].

The relief algorithm is a feature weight algorithm, first proposed by Kira, which gives different weights to each feature, referring to the statistical correlation principle. The advantage of the algorithm is simplicity and high accuracy, while the disadvantage is that it can only deal with binary classification problems [32]. In order to solve the limitation of relief, Kononenko proposed the reliefF algorithm. The steps of the reliefF algorithm are as shown below:

1. Sample set:S, Sampling times:m, Feature set:F, Nearest neighbor sample number: k, Category set:L.
2. Set the output value as the feature weight vector W of each feature.
3. Set the initial values of all feature weights as zero and the feature weight vector W as an empty set.
4. Loop search
  For i=1 to m, do
  Select a sample R from the sample set S randomly;
  For $N \in L$ and $N \ne {\text {Label}}(R)$, do
  Select k nearest neighbor samples ${\mathrm {H}}_{{\mathrm {j}}}$ (j=1,2, ......, k) from the same kind of samples of R and k nearest neighbor samples ${}_{{\mathrm {j}}}$ (j=1,2, ......, k) from the different kind of samples of R;
  End
  End
5. For A=1 to the number of features in feature set F, do
  Update the weights according to the weight formula;
  End

Weight formula:

8 $$\begin{aligned} \begin{array}{c} {\mathrm {W}}({\mathrm {A}})={\mathrm {W}}({\mathrm {A}})-\frac{\sum _{{\mathrm {j}}=1}^{{\mathrm {k}}} {\text {diff}}\left( {\mathrm {A}}, {\mathrm {R}}, {\mathrm {H}}_{{\mathrm {j}}}\right) }{{\mathrm {mk}}}+\frac{\sum _{{\mathrm {c}} \notin {\mathrm {class}}({\mathrm {R}})}\left[ \frac{{\mathrm {p}}({\mathrm {C}})}{1-{\mathrm {P}}({\mathrm {class}}({\mathrm {R}}))} \sum _{{\mathrm {j}}=1}^{{\mathrm {k}}} {\text {diff}}\left( {\mathrm {A}}, {\mathrm {R}}, {\mathrm {H}}_{{\mathrm {j}}}({\mathrm {C}})\right) \right] }{{\mathrm {mk}}} \end{array} \end{aligned}$$

### Support vector machine

SVM is an extensively used machine learning algorithm that refers to statistical learning theory, suitable for small samples, which can account for model complexity and learning ability. The core idea of SVM is to use nonlinear kernel functions to map nonlinear problems, which can not be separated in low-dimensional feature space, to higher-dimensional feature space, and then find a hyperplane in high-dimensional feature space to separate different kinds of samples. Thus, the nonlinear problems that can not be separated in low-dimensional feature space are transformed into the linear problems that can be separated in high-dimensional feature space [33]. The hyperplane requires to separate different kinds of samples and maximize the distance of the target point from the plane, which can be defined as follows:

9 $$\begin{aligned} {\mathrm {H}}: \omega ^{{\mathrm {T}}} {\mathrm {x}}+{\mathrm {b}}=0 \end{aligned}$$

In the process of SVM classification, the choice of kernel function is of capital importance. In this paper, radial basis kernel function (RBF) was selected:

10 $$\begin{aligned} {\mathrm {K}}\left( {\mathrm {x}}_{{\mathrm {i}}}, {\mathrm {x}}_{{\mathrm {j}}}\right) =\exp \left( -\frac{\left\| {\mathrm {x}}_{{\mathrm {i}}}-{\mathrm {x}}_{{\mathrm {j}}}\right\| ^{2}}{2 \sigma ^{2}}\right) \end{aligned}$$

### Experimental setup

The hardware environment is Intel Core i7-9700 CPU@3.60GHz processor, DDR4 32G memory, NVIDIA GeForce RTX 2080Ti 11G graphics card. The Python version is 3.7.3, and the Matlab version is R2020a.

**Table 2:** The sorting results of channels

Classification mode	Channel sort
Mild/Moderate + Severe	2,4,1,9,10,5,6,3,7,11,12,8
Moderate/Severe	4,3,12,7,11,10,8,1,6,5,2,9

**Fig. 4:**
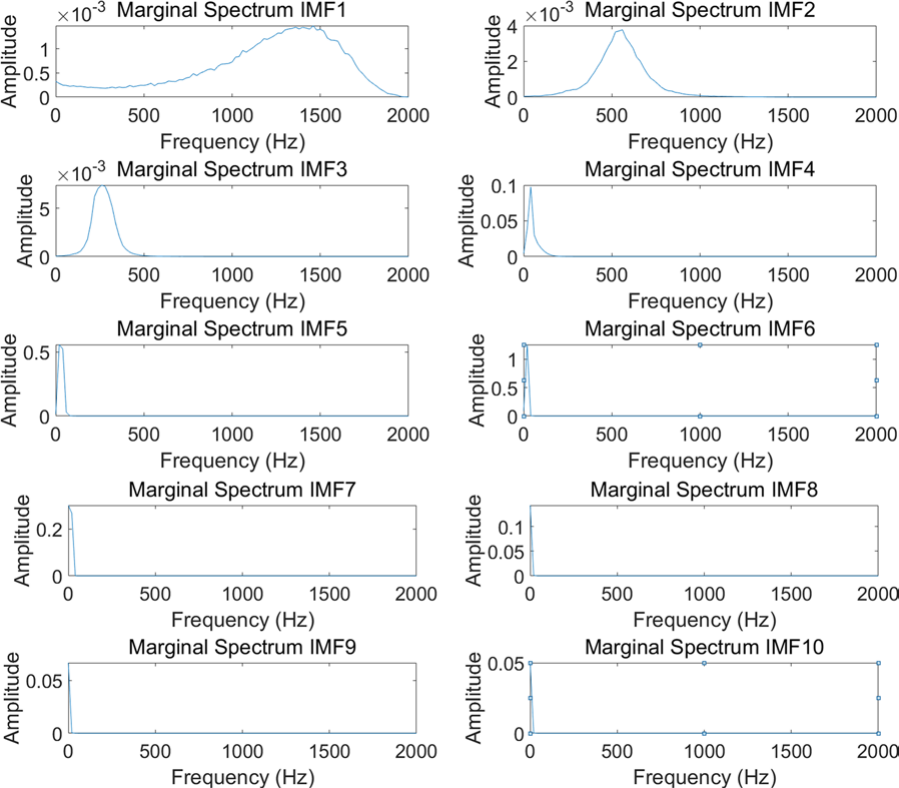
The Hilbert marginal spectrum of IMFs

**Fig. 5:**
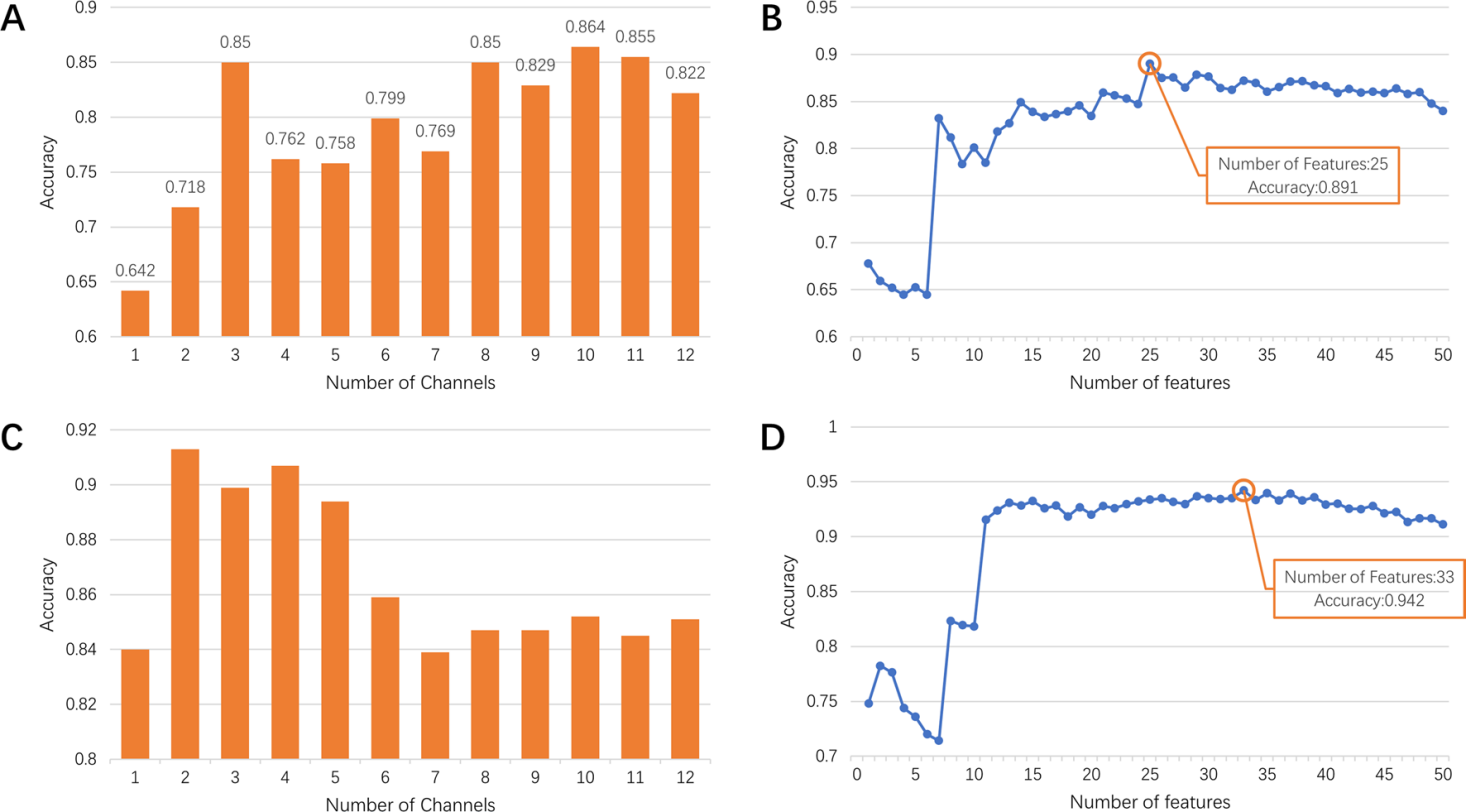
Channels and features selection results. **A** Accuracy of mild COPD and moderate + severe COPD classification using the different number of channels. **B** Accuracy trend of mild COPD and moderate + severe COPD classification using the different feature quantities. **C** Accuracy of moderate COPD and severe COPD classification using the different number of channels. **D** Accuracy trend of moderate COPD and severe COPD classification using the different feature quantities

## Experimental results

The EEMD was used to decompose the pretreated lung sounds. The standard of noise deviation is generally set as 0.2 times the signal standard deviation [31]. Meanwhile, in the case of an appropriate noise level, when the noise number of EEMD is several hundred times, the error caused by residual noise will be less than 1%. Hence, the standard of noise deviation was set as 0.015 and the noise number was 100. In this study, lung sounds were first processed with EMD. It was found that the number of IMFs generated was between 8 and 10. Besides, 80% of lung sounds had 10 IMFs. In EEMD, the number of IMFs is allowed to be specified. Therefore, the IMFs’ number was set to 10, and the remaining IMFs were added to form the residual signal. Figure 3 shows the decomposition result of EEMD. Lung sound is the original signal, IMF1-IMF10 are the 10 IMFs obtained by decomposition, IMF11 is the residual signal.

The Hilbert marginal spectrum of each of IMFs was calculated, and then the HHT-based statistical characteristics were calculated for each IMF, including standard deviation, variance, kurtosis, maximum, median, mode, mean, minimum, energy, and skewness. Figure 4 is the Hilbert marginal spectrum of 10 IMFs for a lung sound signal. The statistical features, a total of 1200, were collected as a dataset.

Standard deviation:

11 $$\begin{aligned} \sigma =\sqrt{\frac{1}{{\mathrm {N}}} \sum _{{\mathrm {i}}=1}^{{\mathrm {N}}}\left( {\mathrm {x}}_{{\mathrm {i}}}-\mu \right) ^{2}} \end{aligned}$$

Variance:

12 $$\begin{aligned} s^{2}=\frac{1}{N} \sum _{i=1}^{N}\left( x_{i}-\mu \right) ^{2} \end{aligned}$$

Energy:

13 $$\begin{aligned} {\mathrm {E}}({\mathrm {t}})=\int _{0}^{{\mathrm {T}}} {\mathrm {H}}(\omega , {\mathrm {t}})^{2} {\mathrm {d}} \omega \end{aligned}$$

Kurtosis:

14 $$\begin{aligned} {\text {Kurt}}({\mathrm {X}})={\mathrm {E}}\left[ \left( \frac{{\mathrm {X}}-\mu }{\sigma }\right) ^{4}\right] \end{aligned}$$

Skewness:

15 $$\begin{aligned} {\text {Skew}}(X)={\mathrm {E}}\left[ \left( \frac{{\mathrm {X}}-\mu }{\sigma }\right) ^{3}\right] \end{aligned}$$

The reliefF algorithm was applied to the feature set of lung sounds. The features with weights less than 0 were eliminated. Then, the weights of respective features were added up to get the total weight of each channel, which was arranged from big to small. Table 2 shows the sorting results of channels under different categories. According to Table 2 in the classification of mild COPD and moderate + severe COPD, the most weighted channels were the 2, 4, 1, and 9, namely L2, L4, L1, and R3 channels. The most weighted channels were 4, 3, 12, and 7 to classify moderate COPD and severe COPD, namely L4, L3, R6, and R1 channels.

By comparing the classification results of SVM using different kernel functions, RBF with strong nonlinear mapping ability was selected, and grid-search was used to optimize penalty factor C and RBF parameter $\gamma$ on the training samples. Finally, C= 10 and $\gamma$=0.2 were selected in the classification of mild COPD and moderate + severe COPD, C=3 and $\gamma$=1.0 in the classification of moderate COPD and severe COPD.

### Mild COPD/moderate + severe COPD

A total of 15 COPD0 and COPD1 samples were taken, labeled as 0. A total of 15 COPD2, COPD3, and COPD4 samples were taken, marked as 1. Features of the different number of optimal channels were selected to form feature sets, and the top 50 optimal features of each feature set were input into SVM, respectively. The train$\_$test$\_$split function of python was used to randomly select 30% of the samples as the test set and 70% samples as the training set. Due to the small amount of data, the test results varied greatly. The average accuracy was obtained by repeated calculations 1000 times to ensure the stability of the results. The results are shown in Fig. 5A.

As shown in Fig. 5A, when the characteristic quantity was set to 50 and the optimal three channels, L2, L4, and L1 channels, were selected, we can already get a high accuracy rate.

The feature set composed of different quantitative features of L2, L4, and L1 channels was input into SVM to test the accuracy, and Fig. 5B was obtained. As shown in the figure, when the number of features is 25, the accuracy rate reaches the highest, 89.13%.

**Fig. 6:**
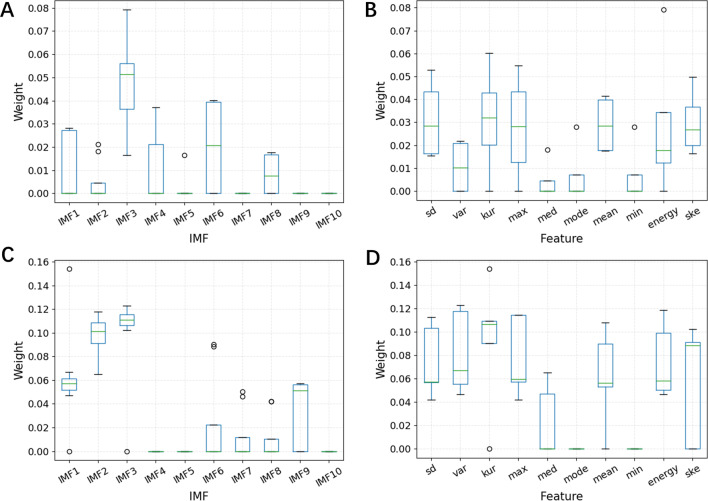
The box plots of IMFs and features under two classification. **A** The box plots of IMFs for mild COPD and moderate + severe COPD classification. **B** The box plots of features for mild COPD and moderate + severe COPD classification. **C** The box plots of IMFs for moderate COPD and severe COPD classification. **D** The box plots of features for moderate COPD and severe COPD classification

Figure 6A, B show the box plots of IMFs and features for mild COPD and moderate + severe COPD classification. According to these figures, we can see that when the 25 features were used to achieve the highest recognition performance, the most responsible feature sets were IMF3 and IMF6, while IMF7, IMF9, and IMF10 did not appear in the feature set at all. For features, the degree of responsibility for standard deviation, kurtosis, maximum, mean, energy, and skewness was similar, while the other features were less responsible. Further observation revealed that the energy, kurtosis, maximum, and standard deviation of IMF3 were the most responsible features.

### Moderate COPD/severe COPD

The analysis focused on 20 subjects. 5 patients with COPD2 and 5 patients with COPD3 were labeled as 0, and 10 patients with COPD4 samples were labeled as 1. Same as the above analysis method, features of the different number of optimal channels were selected to form feature sets, and the top 50 optimal features of each feature set were input into SVM, respectively. The calculations were repeated 1000 times to get the average accuracy, and Fig. 5C showed these results.

As shown in Fig. 5C, when the feature quantity is 50 and the optimal two channels, namely L4 and L3 channels, were selected, we can already get a high accuracy rate. Therefore, we input feature sets composed of L4 and L3 channels into SVM and obtained Fig. 5D by controlling the input feature quantity. It can be seen that when the optimal 33 features of L4 and L3 channels were input, the accuracy rate was the highest, reaching 94.26%.

Figure 6C, D show the box plots of IMFs and features for moderate COPD and severe COPD classification. According to these figures, we can see that when the 33 features were used to achieve the highest recognition performance, the most responsible feature sets were IMF3, IMF2, and IMF1, IMF7, and IMF8 were less responsible, while IMF4, IMF5, and IMF10 did not appear in the feature set at all. For features, the degree of responsibility was similar except that the median value was less responsible, and the mode and minimum value did not appear in the feature set. After further observation, it was found that the most responsible features were the kurtosis of IMF1, the variance and energy of IMF3, and the variance and maximum value of IMF2.

In summary, COPD can be diagnosed only by collecting lung sounds of L1, L2, L3, and L4 channels. The classification of mild COPD and moderate + severe COPD required the optimal 25 features of L2, L4, and L1 channels, with accuracy, sensitivity, and specificity of 89.13%, 87.72%, and 91.01%, respectively. The classification of moderate COPD and severe COPD required the optimal 33 features of L3 and L4 channels, and the accuracy, sensitivity, and specificity were 94.26%, 97.32%, 89.93%, respectively.

**Table 3:** Performance of different machine learning algorithms in the classification of mild COPD and moderate + severe COPD

Model	Accuracy	Sensitivity	Specificity	AUC	F1-Score	Kappa
SVM	89.13	87.72	91.01	96.27	89.13	78.66
	(87.49–91.05)	(85.12–90.48)	(89.64–92.54)	(95.56–96.98)	(87.29–91.03)	(74.99–82.09)
Bayes	84.97	82.61	87.34	93.29	84.65	69.94
	(83.48–86.47)	(80.94–84.29)	(85.32–89.37)	(92.11–94.46)	(83.04–86.26)	(68.33–70.16)
Decision Tree	69.25	66.32	72.24	67.88	68.39	38.52
	(68.33–70.16)	(62.63–70.01)	(67.24–77.23)	(64.69–71.06)	(67.02–69.76)	(36.60–40.46)
DBN	71.74	70.08	73.53	77.75	71.27	43.52
	(66.42–77.06)	(62.99–77.16)	(63.54–83.52)	(73.52–81.98)	(65.92–76.62)	(32.90–54.14)

## Discussion

**Fig. 7:**
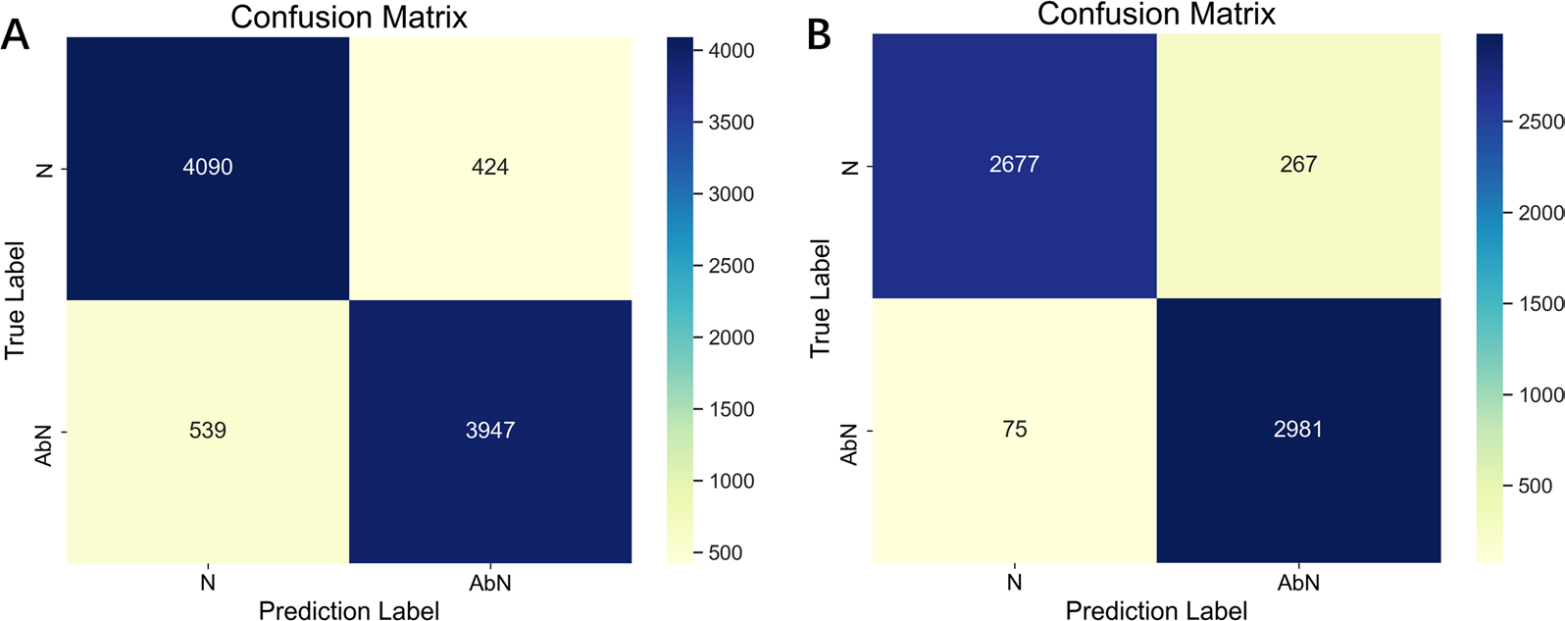
The confusion matrix of SVM under two classifications. **A** The confusion matrix for mild COPD and moderate + severe COPD classification. **B** The confusion matrix for moderate COPD and severe COPD classification

In order to evaluate the classification effect of SVM, we compared the performance of SVM with Bayes, decision tree, and DBN, calculated the confusion matrix of SVM under the two classifications, as shown in Tables 3, 4 and Fig. 7. The classification principle of Bayes is to use the Bayes formula to calculate the posterior probability through the prior probability of an object, and the class with the maximum posterior probability is select as the class to which the object belongs. The decision tree is a tree-like prediction model in machine learning. Internal nodes represent tests on an attribute, each branch represents a test output, and each leaf node represents a category. DBN network is a deep learning network that consists of several unsupervised restricted Boltzmann machines and a supervised backpropagation network. DBN had achieved good results in disease diagnosis by using physiological signals such as electrocardiogram [34], electroencephalogram [35], and lung sounds [19, 20, 28].

**Fig. 8:**
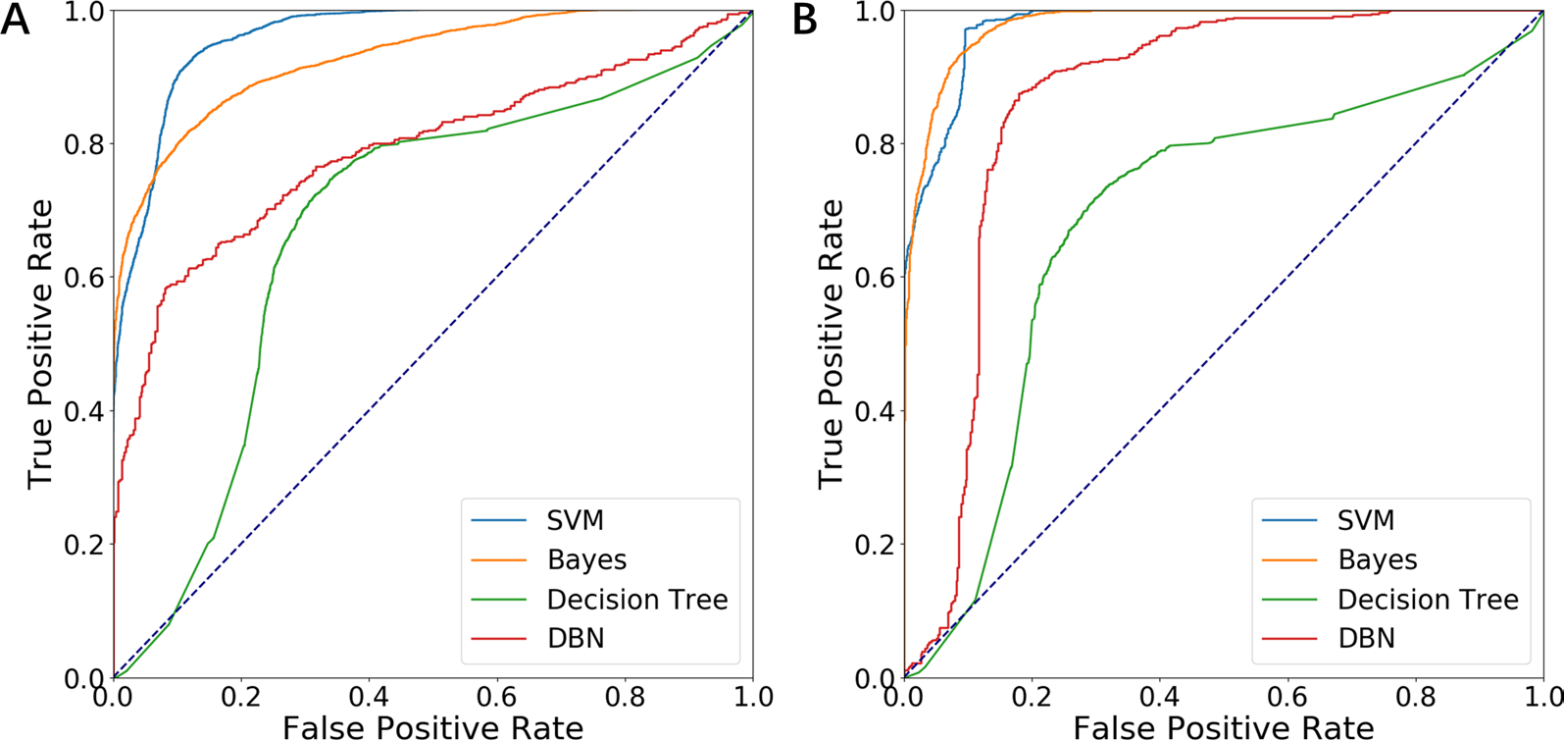
The classification performances of multiple machine learning algorithms. **A** ROC curve under the classification of mild COPD and moderate + severe COPD. **B** ROC curve under the classification of moderate COPD and severe COPD

Bayes and decision tree used the same feature set as SVM, and DBN worked poorly on this feature set. The feature set composed of the optimal 200 features of 12-channel lung sounds worked best through repeated tests. The Bayes model adopted naive Bayes with a prior polynomial distribution, alpha=1.0, and the prior probability was considered. The decision tree model adopted the gradient promotion decision tree, the maximum number of iterations was set as 100, and the learning rate was 1. The DBN iterated 50 cycles, the number of samples for each training was 5, and the learning rate was 0.001. In addition, the output function of DBN was selected as sigmoid. DBN includes an input layer, an output layer, and several hidden layers. In this paper, two hidden layers were used, of which the first layer had 10 neurons and the second layer had 100 neurons, to achieve the highest classification performance. Figure 8 depicts four machine learning algorithms’ receiver operating characteristic (ROC) curves under two classifications.

For small samples, it is not liable to exploit the advantage of the model relied on self-learning. Furthermore, the traditional machine learning algorithm fully reflects that the decision information has more advantages in robustness and expansibility. By comparison, it is observed that although DBN had achieved good results in classifying healthy subjects and COPD patients[18], in this study, the performance of DBN even with the use of full-channel feature sets was far lower than that of SVM. In the two classifications, the classification effect of SVM was superior to Bayes, decision tree, and DBN in terms of accuracy, sensitivity, specificity, Area Under Curve (AUC), F1-score, and Kappa Score. It can also be seen from the confusion matrix that SVM had a good classification effect under the two classifications.

In this study, the weight of each channel was determined by the reliefF channel selection model, and COPD severity was classified by SVM. The study found that the model could obtain a good classification effect by using the 4-channel lung sounds of L1, L2, L3, and L4 channels. The auscultation areas of these 4 channels were all located in the back and the left lung region. For this reason, we consulted much literature and consulted doctors. Although lung auscultation can be performed in both the front chest and back, lung sounds from the back are usually clearer due to the influence of the heart. For the phenomenon that left-side lung sounds can provide more information than right-side lung sounds in COPD patients, no reasonable explanation has been found, and further research is needed.

Previous studies primarily focused on the recognition and classification of abnormal lung sounds. For example, wavelet analysis combined with BP neural network was used to realize automatic recognition of wheezing, crackles, and other abnormal lung sounds [36], and EMD decomposition was used to identify crackles [37]. However, considering that different respiratory diseases may have the same abnormal lung sounds, abnormal lung sounds cannot be used to diagnose COPD severity. For example, COPD is a common cause of wheezing, but asthma, bronchitis, laryngitis may also occur wheezing. Using multi-dimensional features to diagnose COPD has high accuracy and can save diagnosis and treatment costs to some extent [16, 17]. Nevertheless, it is difficult to quickly diagnose the severity of COPD due to a large amount of information required and the need for experienced doctors.

Few studies on COPD diagnosis based on lung sounds focus on separating the digital auscultation records of COPD patients from healthy subjects [18, 20]. Altan et al. investigated how lung sounds could be used to diagnose COPD severity. First, they combined the three-dimensional feature-extraction technique with DBN to classify COPD0 and COPD4 [19]. After that, they used 3D second-order difference plot to extract characteristic abnormalities on lung sounds and then used the deep extreme learning machines classifier to complete the five classifications of COPD severity [21]. Compared with them, our research has the advantage of being more suitable for clinical needs and establishing a channel selection model based on the reliefF algorithm. It reduces the number of features and the calculating pressure, more importantly, reduces the number of channels and simplifies the acquisition process, thus completing the diagnosis of COPD severity more quickly, which helps to improve the portability and practicability of future diagnostic devices, provides a theoretical basis for the advancement of online COPD diagnosis and treatment tools.

It should be noted that despite the rapid development of the application of artificial intelligence in the medical field, due to the lack of standardized datasets, limited by the quantity and quality of data, the models proposed by the current research focus on the innovation of methods. It is not remarkable significant to make a detailed comparison of the quantitative results of these studies from the different sample datasets. It is hoped that a sizeable standardized dataset of lung sounds can be established as soon as possible to accelerate the application of artificial intelligence in the diagnosis of COPD and verify the clinical feasibility of the model.

The clinical diagnosis of COPD is very complicated, which is time-consuming, invasive, and radioactive. Pulmonary auscultation, as an essential part of the diagnosis process, requires experienced respiratory doctors to complete. The proposed model can diagnose the severity of COPD quickly through the 4-channel lung sounds of L1, L2, L3, and L4 channels, which eliminates the long-term clinical examination and saves medical resources.

**Table 4:** Performance of different machine learning algorithms in the classification of moderate COPD and severe COPD

Model	Accuracy	Sensitivity	Specificity	AUC	F1-Score	Kappa
SVM	94.26	97.32	89.93	97.54	94.25	88.16
	(92.70–95.85)	(96.83–98.01)	(87.79–92.16)	(96.96–98.62)	(93.16–95.03)	(85.85–90.42)
Bayes	89.37	99.17	79.61	97.75	90.30	78.74
	(87.70–91.04)	(98.74–99.61)	(76.48–82.73)	(97.06–98.44)	(88.68–91.92)	(75.52–81.96)
Decision Tree	70.63	72.69	68.56	68.04	71.20	41.24
	(69.07–72.18)	(70.13–75.25)	(65.09–72.04)	(66.56–69.52)	(70.17–72.22)	(37.79–44.69)
DBN	83.75	87.80	79.66	85.01	84.42	67.45
	(80.71–86.78)	(83.72–91.87)	(74.25–85.06)	(82.01–88.01)	(81.27–87.57)	(61.40–73.49)

## Conclusion

In this study, firstly, the time-frequency-energy features of 12 channels lung sounds were extracted by HHT; secondly, channels and features were screened by the reliefF algorithm; finally, the feature sets were input into SVM to diagnose COPD severity and compared the performance of SVM with Bayes, decision tree, and DBN. As the experimental results demonstrate, COPD severity can be effectively diagnosed within 5 minutes only by feature extraction, feature selection, and SVM classification of 4-channel lung sounds collected. In the classification of mild COPD and moderate + severe COPD, feature extraction based on HHT took 297.81 s, feature selection based on reliefF algorithm took 0.246s, and SVM classification took 0.836s. In the classification of mild COPD and moderate + severe COPD, feature extraction, feature selection, and SVM classification took 201.97s, 0.238s, and 0.801s, respectively. The noise number of EEMD was set as 100 to reduce the error caused by residual noise, which increased the time. If EMD is used instead of EEMD, only 4.70s and 3.01s are needed for feature extraction under the two categories.

Compared with previous studies on COPD, on the one hand, this model uses the channel selection algorithm, which not only reduces the calculating pressure and prevents over-fitting, more importantly, reduces the difficulty of acquisition and speeds up the diagnosis speed, which is conducive to improving the portability and practicability of future diagnostic devices and promoting the advancement of online diagnosis tools. On the other hand, this study aims to help doctors quickly complete the preliminary diagnosis of mild COPD, moderate COPD, and severe COPD, which is more consistent with the clinical needs and has important clinical significance. The weakest aspect of this research is the quantity and quality of the data, which is currently available exclusively from the RespiratoryDatabase@TR. To better evaluate the reliability of this model in clinical application, we are using a large amount of clinical data in the following research and improving the generalization performance of the model through transfer learning.

## Data Availability

The dataset of lung sounds used in the study is available at https://data.mendeley.com/datasets/p9z4h98s6j/1.
